# Outstanding Response in a Patient With ROS1-Rearranged Inflammatory Myofibroblastic Tumor of Soft Tissues Treated With Crizotinib: Case Report

**DOI:** 10.3389/fonc.2021.658327

**Published:** 2021-06-15

**Authors:** Danila Comandini, Fabio Catalano, Massimiliano Grassi, Guido Pesola, Rossella Bertulli, Antonio Guadagno, Bruno Spina, Matteo Mascherini, Franco De Cian, Federico Pistoia, Sara Elena Rebuzzi

**Affiliations:** ^1^ Medical Oncology Unit 1, IRCCS Ospedale Policlinico San Martino, Genova, Italy; ^2^ Clinic of Medical Oncology, Oncology Institute of Southern Switzerland, Bellinzona, Switzerland; ^3^ Adult Mesenchymal Tumor Medical Oncology Unit, Fondazione IRCCS Istituto Nazionale Tumori, Milan, Italy; ^4^ Anatomic Pathology Unit, IRCCS Ospedale Policlinico San Martino, Genova, Italy; ^5^ Surgical Clinic Unit 1, IRCCS Ospedale Policlinico San Martino, Genova, Italy; ^6^ Department of Surgical Sciences and Integrated Diagnostics (DISC), University of Genova, Genova, Italy; ^7^ Department of Health Sciences (DISSAL), University of Genova, Genova, Italy; ^8^ Department of Internal Medicine and Medical Specialties (Di.M.I.), University of Genova, Genova, Italy

**Keywords:** inflammatory myofibroblastic tumor, inflammatory pseudotumor, sarcoma, ROS1, crizotinib, retreatment, target therapy

## Abstract

Inflammatory myofibroblastic tumor (IMT) is a very rare subtype of sarcoma, which frequently harbor chromosomal rearrangements, including anaplastic lymphoma kinase (ALK) rearrangements (almost 50% of the IMTs) and other kinase fusions such as ROS1. ROS1 fusions are present in about 10% of IMT, almost half of the ALK-negative IMT patients. Apart from radical surgery for resectable tumors, there is no standard-of-care therapy for advanced IMTs. Nonetheless, the use of tyrosine kinase inhibitors has shown promising efficacy in IMT patients with targetable genomic alterations. We report the case of a 24-year-old patient with chemotherapy-refractory metastatic IMT harboring ROS1 kinase fusion, who experienced a significant clinical and pathological response to crizotinib. This clinical case highlights the need to assess all patients with unresectable IMTs for chromosomal abnormalities and gene mutations and address them to targeted agents as well as clinical trials.

## Introduction

Inflammatory myofibroblastic tumor (IMT), also known as inflammatory pseudotumor, is a rare mesenchymal soft tissue tumor characterized by myofibroblastic spindle-cells and a prominent inflammatory infiltrate in an extracellular myxoid/collagenous stroma (1, 2). IMT predominantly affects children and young adults, primarily during the first two decades of life, and occurs mostly in soft tissues and viscera (3, 4). The most common sites of origin are retroperitoneum, abdominopelvic region and lungs (5). Differential diagnosis includes other spindle cell neoplasms such as desmoid fibromatosis, myofibroblastic sarcoma, leiomyosarcoma, gastrointestinal stromal tumor, follicular dendritic cell sarcoma, dedifferentiated liposarcoma and nodular fasciitis (4).

IMT shows a tendency for local recurrence with low risk of distant metastasis and radical surgery is the mainstay of treatment (1, 3, 5). For unresectable or metastatic disease there is no standard-of-care, although many systemic chemotherapeutic regimens have been investigated and showed activity in several trials and case reports (6–14). An overall response rate (ORR) of about 50–60% after first- or second-line treatment was reported in studies on different types of chemotherapy, including anthracycline-based and vinorelbine/vinblastine-based regimens (15, 16).

Almost 50% of the IMTs harbors rearrangements involving the anaplastic lymphoma kinase (ALK), which seem to be associated with localized tumors and better prognosis compared to ALK-negative IMT (17). A substantial proportion of IMTs displays other kinase fusions such as ROS1, PDGFRβ, NTRK3, RET and FN1–IGF1R (18). ROS1 is a receptor tyrosine kinase structurally similar to ALK and ROS1 fusions, mainly YWHAE–ROS1 and TFG-ROS1, are present in about 10% of IMT, almost half of the ALK-negative IMT patients (19, 20).

Given the rarity of IMT, few clinical trials and case series are available on targeted therapy in ALK-positive IMT (21–34). Nonetheless, crizotinib is the only FDA-approved targeted therapy for advanced unresectable ALK-positive IMTs based on the findings of the CREATE study (34).

Moreover, very little data are currently available on the prognosis of ALK-negative IMTs and the role of ROS1 as a driver and potential target in IMT has been documented only in two single cases (17–20).

**Figure 1:**
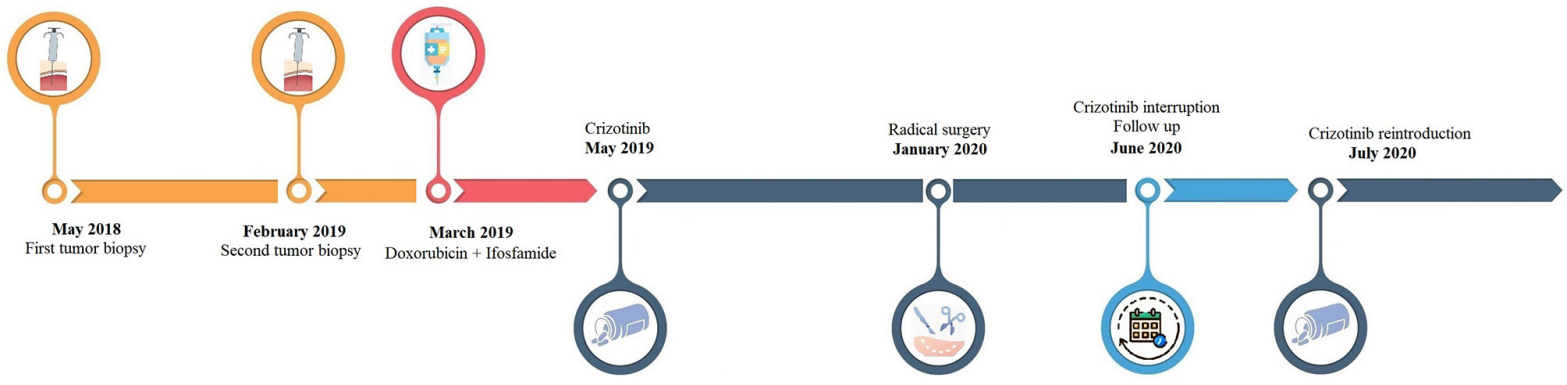
Timeline of patient’s clinical history.

Here we reported the clinical case of an advanced IMT patient harboring a ROS1 rearrangement who extraordinarily responds to crizotinib (**Figure 1**).

## Case Presentation

In April 2018 a 23-year-old Caucasian man presented to the general practitioner for the appearance of a rapidly increasing palpable mass in the proximal part of the left thigh in the last month. His clinal history was negative, as well as his oncological family history. He worked as a plumber and did not report any traumatism. He was a light smoker (less than 5 cigarettes per day for 3 years) and denied the consumption of alcohol or drugs. The clinical examination revealed only a solid lesion of 6 cm of the upper third of the left thigh which was hard, fixed on the deep plane and not painful. A magnetic resonance imaging (MRI) scan was performed, showing an enhancing solid lesion of 50 × 45 × 60 mm localized between the *rectus femoris* and the tensor fasciae latae muscles, associated with enlargement of homolateral inguinal lymph nodes, radiologically suspected for reactive lymphadenopathies, but not clinically palpable for the presence of the main lesion in the proximal part of the thigh.

In May 2018 a biopsy of the mass was performed, revealing an initial diagnosis of nodular fasciitis, and consequently the patient was addressed to follow up. In consideration of the clinical dimensional increase of the mass, a new MRI was performed in July 2018 confirming the increase of the left thigh lesion (54 × 64 × 75 mm). A surgical approach was, therefore, planned but the pre-operative blood tests reported an important elevation of serum transaminases values (GOT = 145 U/L, GPT = 440 U/L, GOT/GPT = 0.32). According to the suspicion of liver metastases, a staging Total Body (TB) computed tomography (CT) scan was performed in November 2018, but no secondary lesions were shown. According to the hepatological consultation, the transaminase elevation was not amenable to a viral or autoimmune disease, but a paraneoplastic syndrome.

The radical surgery was, therefore, planned in February 2019 but the pre-operative 18F-FDG-PET revealed, in addition to the main tight lesion (SUV max 23), the presence of two paravertebral lesions (SUV max 21), suspected for metastases, and a minimal uptake of the homolateral inguinal lymph nodes (SUV max 2.4), questioning the initial diagnosis of nodular fasciitis.

In consideration of the different uptake compared with the main lesion, the inguinal lymph nodes were considered reactive lymphadenopathies.

A new biopsy was performed in February 2019 with the diagnosis of myofibroblastic sarcoma. Considering the complexity of the case, in agreement with the patient, a second opinion from an expert pathologist was asked by the first hospital where the patient was followed.

**Figure 2:**
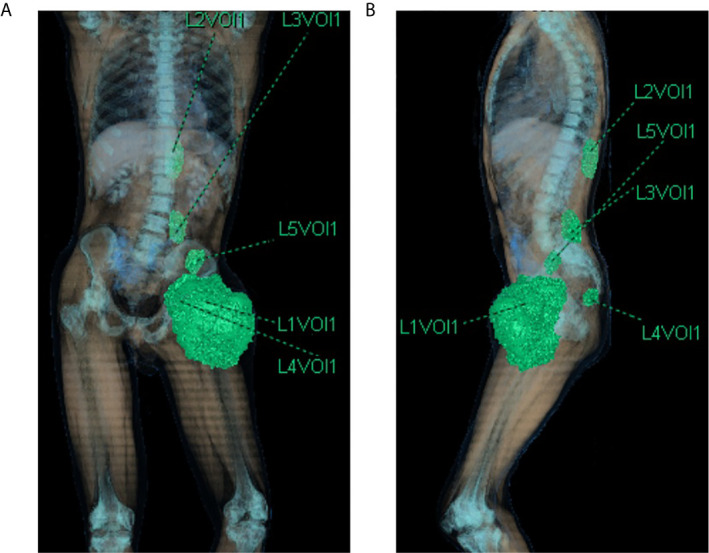
CT scan 3d-reconstruction of the baseline tumor lesions **(A)** coronal plane; **(B)** sagittal plane). L1Vol1 **=** left thigh mass; L2Vol1 = lesion of the left dorsal muscles next to D11 vertebral body; L3Vol1 = lesion of the left dorsal muscles next to L5 vertebral body; L4Vol1 = lesion of the left gluteus muscles; L5Vol1 = lesion of the left iliopsoas.

**Figure 3:**
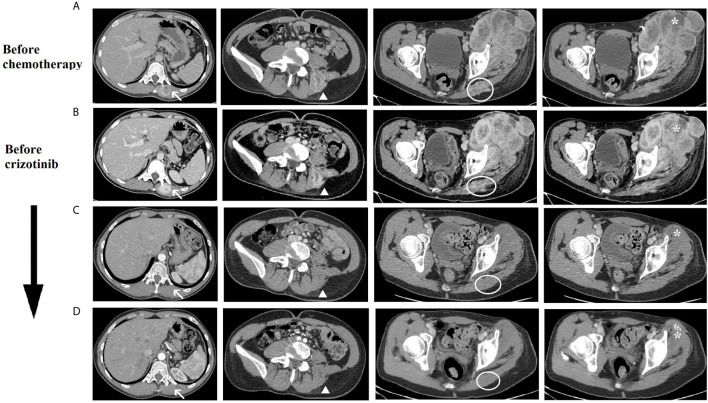
CT scan imaging of the tumoral lesions during patient’s treatment history. Evolution during treatment of the tumoral lesions: lesions of the left dorsal muscles next to D11 vertebral body (arrow) and L5 vertebral body (arrowhead), lesion of the left gluteus (circle) and left thigh mass (asterisk). **(A)** before chemotherapy (March 2019), **(B)** after second cycle of chemotherapy and before crizotinib (May 2019), **(C)** after 4 months of crizotinib (September 2019), **(D)** after 7 months of crizotinib (December 2019).

The TB CT scan of March 2019 showed the left thigh mass of 170 × 135 mm, a similar mass in the context of the left iliopsoas (40 × 25 mm) and gluteus muscles (19 × 21 mm), other two tumoral nodules in the dorsal muscles next to D11 vertebral body (37 × 30 mm) and L5 vertebral body (40 × 30 mm) (**Figures 2A, B** and **3A**). The reactive inguinal lymphadenopathies were not viewable.

**Figure 4:**
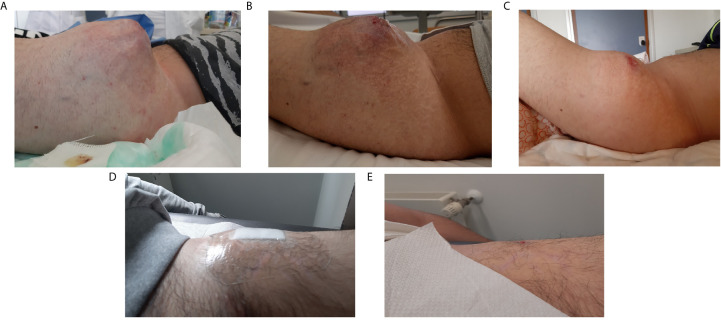
Clinical presentation of the left thigh mass during patient’s treatment history. **(A)** before chemotherapy (March 2019), **(B)** after second cycle of chemotherapy and before crizotinib (April 2019), **(C)** after 7 days of crizotinib, **(D)** after 3 months of therapy (August 2019), **(E)** after 7 months of therapy (December 2019).

In the same month, in consideration of the rapid progression of the primary lesion (**Figure 4A**) and the final diagnosis of sarcoma, the start of standard chemotherapy with doxorubicin and ifosfamide was indicated. Only at this point, the patient presented to our cancer department for the start of the chemotherapy. The patient generally did not tolerate the chemotherapy due to the onset of severe hematological toxicity (grade 4 neutropenia according to Common Terminology Criteria for Adverse Events (CTCAE) v5.0) but a normalization of serum transaminases values (GOT 12 U/L and GPT 16 U/L) was observed. The physical examination showed a dimensional stability of the main tight lesion. In April 2019 the patient started complaining of dorsal pain and we, therefore, opted for palliative radiotherapy of the dorsal lesions (20 Gy in two fractions).

Between the second and the third cycle of chemotherapy, the final histological diagnosis of IMT was provided by the second opinion of the external expert pathologist. Immunohistochemistry (IHC) for the assessment of ALK was performed but the result was negative. A next-generation sequencing (NGS) of the tumoral tissue was subsequently performed and revealed the presence of the YWHAE1-ROS1 fusion (images not available).

According to the absence of clinical and radiological response to chemotherapy (**Figures 3B** and **4B**), the presence of this rare targetable mutation and the patient’s preference to stop chemotherapy, the patient started crizotinib (250 mg orally, twice daily) as off-label therapy in May 2019. After only 7 days of treatment, we observed a significant dimensional decrease of the left thigh mass (**Figure 4C**). Despite the good clinical response, the patient developed visual disturbances (blurred vision), which is a common adverse event reported with crizotinib (34). In consideration of the mild grade of the adverse event and the rapid clinical benefit, the treatment with crizotinib was continued with no dose reduction. The visual symptoms spontaneously resolved after the first month of therapy.

In August 2019, after 3 months of therapy, an outstanding clinical response was observed (**Figure 4D**). The CT scan of September 2019, also, showed a significant dimensional decrease of the left thigh mass (40 × 30 mm) and a complete regression of the lesions of the iliopsoas, gluteus and dorsal muscles (**Figure 3C**).

The patient experienced also a significant clinical benefit with the disappearance of dorsal pain and the functional recovery of the left leg with the resumption of normal daily activities.

In December 2019, after 7 months of therapy, an additional minimal shrinkage of the tumor mass was observed (38 × 30 mm) (**Figure 3D**). In January 2020, after 8 months of treatment, in consideration of the outstanding clinical and radiological response and the patient’s preference to remove all the visible disease, the patient underwent wide excision of the residual tight lesion (**Figure 4E**).

**Figure 5:**
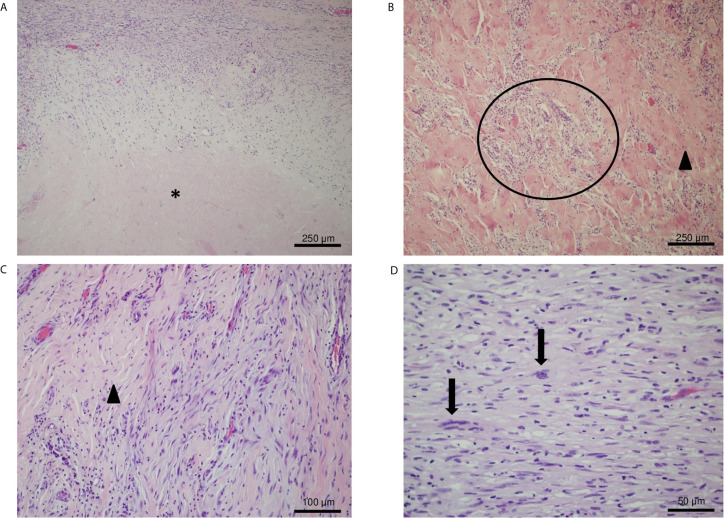
IHC analysis of the resected tumor. **(A)** Hematoxylin and eosin (H&E) stain, original magnification 10×: necrotic area (asterisk) and sclerotic tissue with residual neoplastic spindle cells. **(B, C)** H&E stain, original magnification 20×: foamy histiocytes (circle) and a vascularized fibrotic area of connective tissue (arrowhead) mark the tumor bed. **(D)** H&E stain, original magnification 40×: atypical spindled neoplastic cells set in a loose collagenous matrix with a mild inflammatory infiltrate. Neoplastic cells show hyperchromatic and enlarged nuclei (arrows).

The histological examination showed a large tumor bed composed of necrotic area, sclerotic fibrous tissue and a mixed chronic inflammatory infiltrate with abundant foamy histiocytes. Within the tumor bed, two residual foci of neoplasia were observed (both about 5 mm in diameter). The neoplastic foci displayed spindle-shaped cells embedding in a loose myxoid stroma (**Figures 5A–D**) (TNM: ypT1, according to UICC—VIII ed. 2017).

In consideration of the initial advanced stage of the disease, crizotinib was resumed in February 2020, two weeks after radical surgery. In consideration of the absence of scientific evidence on the efficacy of crizotinib in the “adjuvant” setting and the patient’s preference to stop any oncological treatment, crizotinib was continued until June 2020 when the patient reached the completion of a full year of treatment. Moreover, the patient agreed with this clinical decision of treatment discontinuation also to reduce the hospital accesses during the SARS-CoV-2 pandemia.

**Figure 6:**
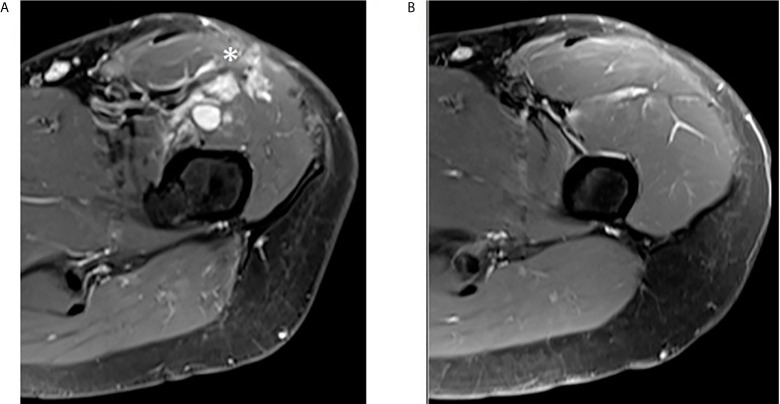
Complete regression of multifocal local recurrence in the left thigh. **(A)** Post-contrast MR T1 fat saturated axial image of upper left thigh shows some irregular enhancing nodules in the anterior compartment (asterisk). **(B)** The same sequence obtained three months later in the same area reveals complete nodules disappearance.

In July 2020, the first radiological assessment off-therapy (2 months) the TB CT scan and the subsequent MRI (**Figure 6A**) showed a multifocal local recurrence in the left thigh without evidence of new distant metastasis. Therefore, in August 2020 we decided, in agreement with the patient, to resume the treatment with crizotinib. After 3 months of therapy (November 2020) the TB CT scan and the MRI (**Figure 6B**) showed a complete regression of the disease.

*In April 2021 the patient is still on treatment with crizotinib*, in good clinical conditions and without symptoms or clinical or radiological evidence of disease.

## Discussion

The histopathological diagnosis of rare tumors, especially sarcomas, is one of the most difficult tasks to get to, but also one of the most important ones as it may change their multidisciplinary approach. We know from the literature that the initial histological diagnosis often needs to be reviewed by an expert pathologist because in about 15% of cases major discordances are found (35).

In our case, we got to the final diagnosis only after the expert pathology revision and after almost one year from the initial biopsy. IMT is indeed an ultra-rare sarcoma whose diagnosis can be very hard, both for clinicians and pathologists, also considering the multiple differential diagnosis (4).

When the clinical and morphological aspects are compatible with the IMT diagnosis, the immunohistochemical assessment of ALK-staining is paramount for diagnosis as its positivity is observed in 50–60% of IMT cases. In case of a positive result (ALK-positive by IHC), the experts suggest performing FISH analysis for the evaluation of ALK fusions (17, 36). In case of negative IHC result, FISH analysis for the detection of ROS1 and NTRK3 should be performed considering that new target therapies are recently available (20). NGS may be more appealing in this scenario because can also detect other gene fusions characteristic for IMT such as PDGFR, RET, FN1–IGF1R and other rarer mutations, that may be possible targetable mutations in ALK/ROS1-negative IMTs (18, 20, 37, 38). The molecular assessment of IMTs is, therefore, crucial to choose the best target treatment (15, 16).

In 2010 the first case of ALK-positive IMT responding to crizotinib was reported by Butrynski et al., as compared with no response observed in an ALK-negative IMT patient receiving the same treatment (21). Other case series and phase I/II trials reported positive activity and efficacy results of crizotinib in ALK-positive IMTs, which are increased when compared with ALK-negative IMTs (22, 23, 30, 34).

The CREATE trial was the most important study assessing the activity and safety of crizotinib in advanced or inoperable IMTs with and without ALK alterations (34). ALK-positive patients (n = 12) reported an objective response rate (ORR) of 50%, including 17% complete responses, with a disease control rate (DCR) of 100%. On the other hand, ALK-negative patients (n = 7) reported lower ORR and DCR (14 and 86% respectively). Also, survival rates were higher in ALK-positive IMT patients: progression-free survival at 1-year and 2-year (1-yr/2-yr PFS) was of 73.3 and 48.9% in ALK-positive patients compared to 53.6 and 35.7% in ALK-negative ones.

These data suggested the use of crizotinib only in the ALK-positive population, but it is important to notice that the mutations in the ALK-negative group were not reported and the number of patients was very small.

The first case of a ROS1-positive pulmonary IMT pediatric patient responding to crizotinib was reported by Lovly et al. (20). Similarly, a long-lasting remission with crizotinib was observed in another ROS1-rearranged pulmonary pediatric IMT reported by Mai et al. (39).

In consideration of these promising results and those observed in ROS-1-positive non-small cell lung cancer patients, we decided to propose crizotinib to our patient. This clinical case is the first case reported in the literature on an adult patient with a ROS1-rearranged IMT of soft tissues who experienced an outstanding clinical and radiological response to crizotinib.

However, this clinical case is not interesting only for the rarity of the tumor and the exceptional treatment response, but also for the different clinical points in the decision-making of such a rare tumor. Considering the evident limitation due to the absence of literature data, many clinical concerns were raised during the clinical management of the patient. Every diagnostic and therapeutic choice has been taken considering these limits and was previously discussed with the patient.

After the excellent clinical response to crizotinib, we opted for wide excision after 8 months of therapy for the following different reasons. Neoadjuvant or adjuvant therapies for IMT patients have been reported in few clinical cases and seemed a feasible approach (40–42). The only data on crizotinib treatment in ALK-negative patients came from the phase II trial CREATE: in these patients the median duration of response was 7.6 months (34), so that we could not exclude a possible progression of disease in a short time continuing the targeted therapy. Moreover, this decision was guided by the patient’s desire to undergo surgery because of his fear of the local disease that impacted his quality of life.

In the after-surgery scenario, the main problem was to decide whether to resume the treatment with crizotinib even in the absence of evident disease or not. In the literature, very few reports have been written on neoadjuvant or adjuvant use of targeted therapy or chemotherapy in IMTs (40–42). Nevertheless, the impressive response to crizotinib observed in our patient and the initial metastatic disease led us to continue therapy after surgery, even after an almost complete pathological response.

The key point then was to determine for how long this therapy should be continued. A recent retrospective study published by Trahair et al. reported eight cases of ALK-positive IMT children and adolescents treated with crizotinib and surgery with different stages of disease (42). The median duration of treatment with crizotinib, either in the adjuvant or neoadjuvant setting, was 1 year with most patients still disease-free at a median follow up of three years (42). This study revealed that different strategies were all justified and, after treatment discontinuation, most patients maintained the response achieved. These data, even if weak, in addition to the patient’s preference, lead us to discontinue treatment after the completion of one year of treatment.

At disease progression, after therapy discontinuation, we decided to resume crizotinib because, as reported in the literature, new responses are still possible and, moreover, in this rare disease other effective therapies are still not approved and difficult to get the access (25–29, 32, 33, 42–45).

In fact, in ALK-positive IMT patients, after progression to crizotinib, different ALK inhibitors, such as ceritinib, alectinib and lorlatinib, have been investigated reporting promising responses (25–29, 32, 33, 42–45).

Also in ROS1-rearranged patients, especially those with NSCLC, other ROS1 inhibitors (lorlatinib, entrectinib, repotrectinib) have been investigated and may be a possible future therapeutic option in ROS1-positive disease, including IMT (46–50).

In this scenario, molecular assessment is recommended in ALK-negative IMT patients to identify potentially actionable mutations for which new target therapies are available (36). Further clinical trials and expanded access programs are, therefore, needed to address rare tumors, like IMT, to specific and effective treatments.

## Data Availability Statement

The original contributions presented in the study are included in the article/supplementary material. Further inquiries can be directed to the corresponding author.

## ETHICS STATEMENT

Written informed consent was obtained from the patient for the publication of any potentially identifiable images or data included in this article.

## Author Contributions

DC, FCa, and SER: conception, design, writing and review of the manuscript. FCa, MG, and GP: draft of the manuscript and analysis of the patient’s data. RB, MM, FDC, and FP: clinical management of the patient and provision of patient’s data. AG and BS: elaboration and analysis of the pathology. All authors contributed to the article and approved the submitted version.

## Conflict of Interest

The authors declare that the research was conducted in the absence of any commercial or financial relationships that could be construed as a potential conflict of interest.
